# A gene expression profile of the developing human retinal pigment epithelium

**Published:** 2012-12-15

**Authors:** Zhongyu Zhang, Yan Zhang, Huizhen Xiao, Xiaolei Liang, Dawei Sun, Shaomin Peng

**Affiliations:** 1 Department of Ophthalmology, the Second Affiliated Hospital of Harbin Medical University, Harbin, China; 2 Department of Ophthalmology, Inner Mongolia Forestry general Hospital, Yakeshi, Inner Mongolia, China

## Abstract

**Purpose:**

The molecular mechanisms associated with human retinal pigment epithelium (RPE) development constitute the basis for cell replacement therapy for the treatment of retinal degenerative diseases. In the current study, gene expression analysis of the human fetal RPE during development was performed and was compared with the human native RPE.

**Methods:**

Microdissection of the human RPE at three time points (13 weeks and 16 weeks of gestation and in mature adult eyes) was performed, and total RNA was isolated. Equal amounts of RNA were pooled from two or three independent donor eyes for each time point in each group. Gene expression was analyzed by hybridization to microarray chips. Validation was accomplished by comparing the microarray expression proﬁles with quantitative real-time reverse transcriptase-polymerase chain reaction (qRT^2^-PCR) analysis of selected genes and by comparing selected expression proﬁles with predicted proﬁles based on previous studies.

**Results:**

Of the 45,033 probe sets on the microarray, 30,736 were detected. A total of 3,498 differentially expressed genes could be clustered into eight patterns of expression that were statistically significant. Analysis of the expression patterns of genes coding for key functions (pigment synthesis, visual cycle, phagocytosis, adherens and tight junctions, and transcellular transport) indicated that the human RPE achieves a high degree of maturity during early pregnancy. Compared with 154 signature genes in the RPE, 148 candidate genes were identified in this study, including 53 downregulated genes and 5 upregulated genes. The qRT^2^-PCR results showed similar expression trends to those obtained by microarray analysis at the three time points.

**Conclusions:**

This study demonstrated gene expression profiles in the human RPE during normal development. These ﬁndings indicate that the human RPE has different expression patterns than those of other animals. The results of this study may be helpful in furthering the understanding of the developmental processes occurring in humans and of the differentiation of RPE cells derived from human embryonic stem cells and from human induced pluripotent stem cells.

## Introduction

The retinal pigment epithelium (RPE) cells in human eyes form a quiescent, polarized epithelial monolayer located between the neural retina and the vascular choroid, and these cells serve to support and maintain the photoreceptor cells and other outer retinal cells via multiple mechanisms, including formation of the blood-retinal barrier, absorption of stray light, supply of nutrients to the neural retina, and regeneration of visual pigment, as well as the uptake and recycling of the shed outer segments of photoreceptors [1]. Because of its important function in supporting photoreceptors, dysfunction and loss of the RPE leads to photoreceptor degeneration or apoptosis. Substantial evidence supports the notion that the dysfunction and death of RPE cells play a critical role in the pathogenesis of age-related macular degeneration (AMD) [2-4], which is the leading cause of blindness among the elderly in the developed world. As the population continues to age, the number of people in the United States with advanced AMD is expected to exceed 2.9 million by 2020 [5].

The emerging strategy of cell replacement therapy has provided a new approach to the treatment of AMD. Various types of dissociated RPE cells, such as immortalized adult RPE cell lines, human fetal RPE cells, and RPE cells derived from human embryonic stem cells (hESCs) and human induced pluripotent stem cells (hiPSCs), have been transplanted into the subretinal space in animal models of retinal degeneration caused by dysfunction of the RPE [6-13]. Moreover, Steven Schwartz reported on the first set of phase I clinical trials, which are ongoing, in which two patients were treated with RPE cells derived from hESCs [14]. Although many of these studies have demonstrated the protection of photoreceptors and even improvement in visual function after transplantation, the visual protection and improvement have been found to be different due to the differential sources of cells; for example, fetal RPE cells may yield better results than adult RPE cells [15]. Nevertheless, most studies have shown that transplanted cells die within 2 weeks to several months and that long-term survival is not achieved [12,13,16]. Thus, we are still faced with major obstacles to such cell-based therapies in clinical application. In addition, Liao’s studies showed that only 42 genes among 108 selected RPE signature genes are commonly shared by human fetal RPE, hESC-RPE, and hiPSC-RPE cells [17]. Moreover, rather than a polygonal monolayer structure, which is characteristic of a functional RPE that is formed after transplantation, it is common to find clumps of cells that fail to integrate with the host monolayer.

Therefore, the culturing ofmore “in vivo like” RPE cells and the integration of these transplanted cells into the host tissue in a diseased environment are still significant challenges. To solve these problems, we must first determine the molecular mechanisms of human RPE development in its native physiologic environment. To date, most of our understanding regarding mammalian RPE development has been derived from studies in rodents [18]. Genomic analyses of RPE development have been reported in zebrafish [19], chickens [20,21], and humans [22]. Although the results from animal RPE developmental studies are invaluable, however, they are not always transferrable to humans.

In the present study, we analyzed thousands of genes expressed in the different stages of the fetal and adult RPE using microarray and quantitative real-time reverse transcriptase–polymerase chain reaction (qRT^2^-PCR) analysis. These RPE gene expression profiles, combined with a bioinformatics analysis, enabled us to better understand the changes in gene expression and the intrinsic program of gene regulation that accompany human RPE development. Additionally, we expect that this research will contribute in the future to the optimization of cell replacement therapy and the differentiation of hESCs and hiPSCs.

## Methods

### Isolation of the retinal pigment epithelium

This research followed the tenets of the Declaration of Helsinki on the use of human material for research and written informed consent was obtained before sample collection. Fetal human eyes (13 and 16 weeks of gestation) and adult eyes (40–60 years old), obtained from Harbin Medical University (Harbin, China), were dissected to obtain the posterior eye cups. After the retinas were carefully peeled off, the eye cups were treated with 2% dispase (Invitrogen-Gibco, Grand Island, NY) in PBS at 37 °C for 20 min. The RPE sheets were then gently peeled from the choroid with fine forceps under a dissecting microscope, and the sheets were stored in RNAlater (Qiagen, Valencia, CA).

### RNA extraction and microarray

Equal amounts of RNA were pooled from the three independent replicates used for each time point in each group. RNA was extracted using TRIzol (Invitrogen, Carlsbad, CA) and the RNeasy kit (Qiagen), according to the manufacturer’s instructions. The concentration and quality of RNA were determined using a bioanalyzer (Agilent Technologies, Palo Alto, CA) and/or a nanodrop ND-1000 spectrophotometer (Thermo Fisher Scientific Inc., Wilmington, DE). All of the samples had A_260_ /A_280_ ratios of total RNA of 2.0, and the ratio of the 28S to 18S rRNA bands was greater than 1.8.

Approximately 5 μg of total RNA in each sample set (pooled separately fromthree 13-week fetal eyes, two 16-week fetal eyes, and two adult eyes) was amplified, labeled using a NimbleGen One-Color DNA Labeling Kit (NimbleGen Systems, Madison, WI), and hybridized using the NimbleGen Hybridization System (NCBI HG18, Build 36; 12×135k Array, Inc., Madison, WI). The NimbleGen System contains 45,033 probe sets. After hybridization and washing, the processed slides were scanned with the Axon GenePix 4000B microarray scanner.

### Data processing

Raw data were extracted as pair files using NimbleScan software (version 2.5). The NimbleScan software’s implementation of robust multichip averaging offers quantile normalization and background correction. The three gene summary files were imported into Agilent GeneSpring software (version 11.0) for further analysis. Genes that had values greater than or equal to the lower cutoff—100.0 in all samples (“All Targets Value”)—were chosen for data analysis. Differentially expressed genes were identified using a combination of t-statistics, false discovery rates, and fold-change screening. Spots with twofold or more differences in intensity between any two groups were marked as differentially expressed genes. The gene expression patterns were analyzed using Short Time-series Expression Miner (STEM, version 1.2.2b) software. Gene ontology (GO) analysis was performed using the standard enrichment computational method. Data were deposited in the Gene Expression Omnibus database, accession # GSE40980.

### Quantitative real-time reverse transcriptase–polymerase chain reaction

**S**even genes that exhibited significant differential expression profiles based on cDNA microarray analysis were chosen for further validation by two-step qRT^2^–PCR analysis. Total RNA was extracted using an RNeasy Mini Kit (Qiagen). First-strand cDNA was synthesized from a 2 µg aliquot of the total RNA samples using the oligo-dT primer and MMLV reverse transcriptase (Epicenter, Madison, WI). Real-time RT–PCR was performed with a Rotor-Gene 3000 cycler (Corbett Research) and SybrGreen I (Invitrogen) according to the manufacturer’s protocol. The running conditions were as follows: 5 min at 95 °C, followed by 40 cycles of 10 s at 95 °C (denaturation), 15 s at 59 °C (annealing), and 20 s at 72 °C (extension). The experiments were performed in triplicate with a minimum of two biologic repetitions. Glyceraldehyde-3-phosphate dehydrogenase was used as a control to normalize the data. A total of 40 cycles were run, and the *C*(*t*) values were used to evaluate the relative expression levels of the tested genes. Standard curves were generated for each gene. To determine the specificity of DNA quantification, a melting curve was generated for each gene. Specific primers were designed with Primer software (version 5.0, Integrated DNA Technologies, Skokie, IL). The primers used for qRT^2^-PCR are listed in Table 1.

**Table 1:** Primers Used for qRT^2^-PCR.

Gene	Ref Seq ID	upstream	Downstream	Size (bp)
*GAPDH*	NM_002046	5′GGGAAACTGTGGCGTGAT3′	5′GAGTGGGTGTCGCTGTTGA3′	299
*RPE65*	NM_000329	5′GATGCCTTGGAAGAAGATGAT3′	5′CCATGAAAGGTGACAGGGAT3′	155
*SFRP5*	NM_003015	5′GGGATTCATTCCTCAGCCTCT3′	5′ACCCTGCATGTATTGGTTGTCTA3′	98
*GPR143*	NM_000273	5′CCCCATATTCCTCAGACTCAAC3′	5′GCATGAACCCTTTCTCCTATCCT3′	159
*TYRP1*	NM_000550	5′GATTCCACTCTAATAAGCCCAAAC3′	TTCCAAGCACTGAGCGACAT3′	198
*CDO1*	NM_001801	5′ATCCATACTAGGAGCGTCAGTG3′	5′TTAGTCCTCCAGGTAACAAAGC3′	131
*CHRNA3*	NM_000743	5′CAATCACGCTGGGAATAGGT3′	5′CCATAGTGCTAAGGCTGAGAAAC3′	102
*CLDN19*	NM_148960	5′ACGGTGGCTCACGCTTGTA3′	5′AGTGGCACGATCTTGGCTG3′	236

## Results

### Analysis of general gene expression in the fetal and adult retinal pigment epithelium

To generate gene expression profiles during human RPE development, three time points were chosen: 13 weeks and 16 weeksof fetal development and mature adulthood (40–60 years old). The cDNA microarrays with 45,033 probes were hybridized with targets made from the mRNA (mRNA) of the RPE of 13-week-old fetuses, 16-week-old fetuses, and adults. The hybridizations were normalized by internal controls on each membrane, and comparisons were made among membranes. Of the 45,033 probe sets on the microarray, 30,736 were detected. Among these positively expressed genes, 242, 4,245, and 4,336 genes demonstrated twofold or more differences in signal intensities, either up- or downregulated, between 13-week and 16-week fetal RPEs, between 13-week and adult RPEs, and between 16-week and adult RPEs, respectively.

### Gene expression profile clustering

**Figure 1:**
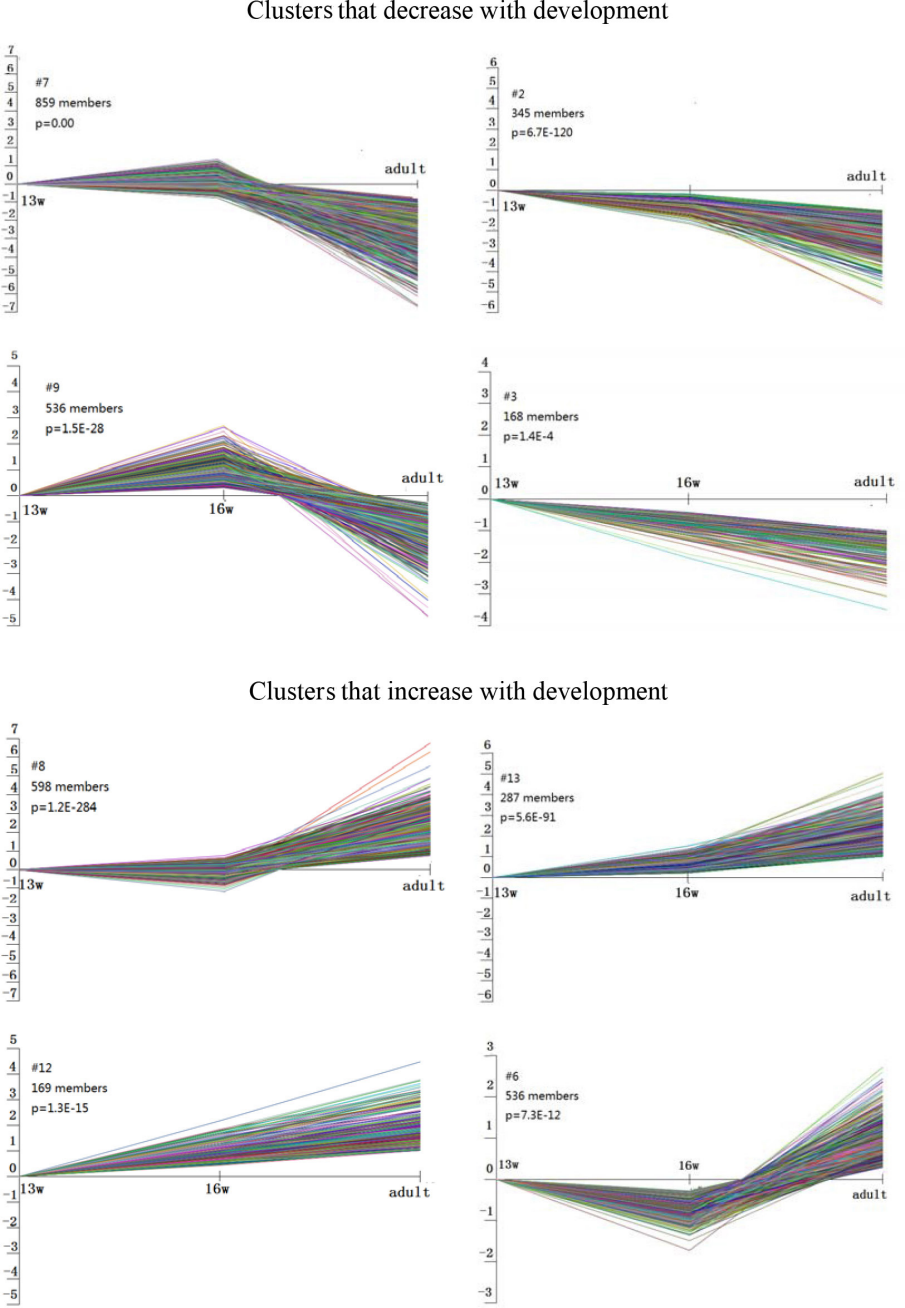
Cluster analysis of differentially expressed genes. Probe sets were grouped by STEM software into eight clusters that were statistically significant. The notations “13 w,” “16 w,” and “adult” on the x-axis represent different time points. The numbers at the upper right indicate the number of genes belonging to each cluster and the p value significance.

Gene cluster analysis was performed using STEM version 1.2.2b software. STEM implements a novel clustering method that is based on a set of distinct and representative short temporal expression profiles, where each probe in the data set is assigned to the profile with the closest match. The genes expressed during RPE development could be classified into 16 clusters, and 8 were judged to be statistically significant. These 8 clusters included 3,498 probe sets. As shown in Figure 1, clusters 2 and 3 and clusters 12 and 13 represented the genes with consistently decreased expression and consistently increased expression throughout development, respectively. Clusters 7 and 9 represented the genes that were expressed at low levels at 13 weeks of gestation and in adulthood but that were upregulated in the 16-week-old fetus. In contrast to cluster 6, cluster 8 represented genes that were expressed at high levels in the 13-week-old fetus and in adulthood but were downregulated in the 16-week-old fetus. Genes with consistently decreasing expression during retinal development were more common than genes with consistently increasing expression. Gene ontology analysis revealed that these differentially expressed genes were associated with, for example, differentiation, signal transduction, protein translation, metabolism, DNA binding, and transcription.

### The expression patterns of specific groups of genes

**Figure 2:**
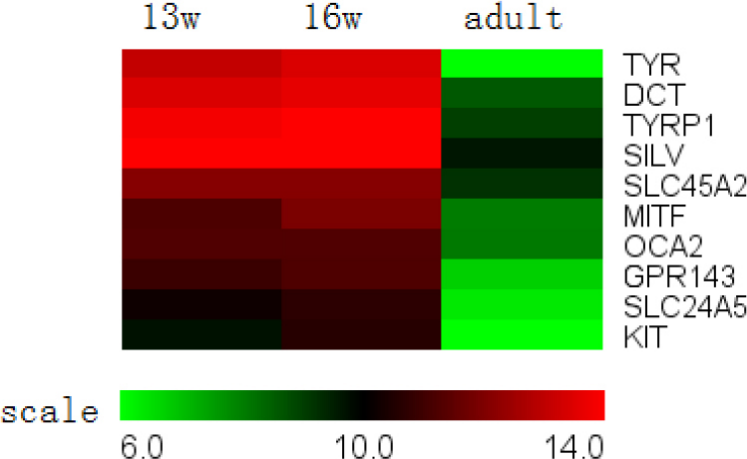
Pigment synthesis genes expressed in the retinal pigment epithelium. All mRNA expression of genes associated with pigment synthesis proteins decreased throughout development.

**Figure 3:**
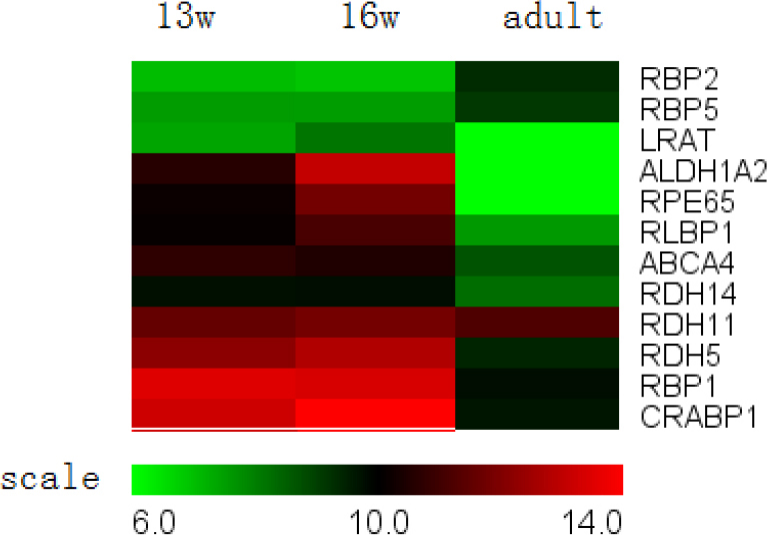
Visual cycle genes expressed in the retinal pigment epithelium. Some mRNAs (mRNAs) for visual cycle proteins were stably expressed during development, including *ABCA4*, *RBP1*, *RBP2*, *RBP5*, *RLBP1 (CRALBP)*, *CRABP1*, and *RDH11*. Others showed minimal changes between 13- and 16-week-old fetuses, but a significant decrease was observed between 16-week-old fetuses and adults; these included *RPE65*, *LRAT*, *ALDH1A2*, *RDH5*, and *RDH14*. *IRBP* was not detected.

**Figure 4:**
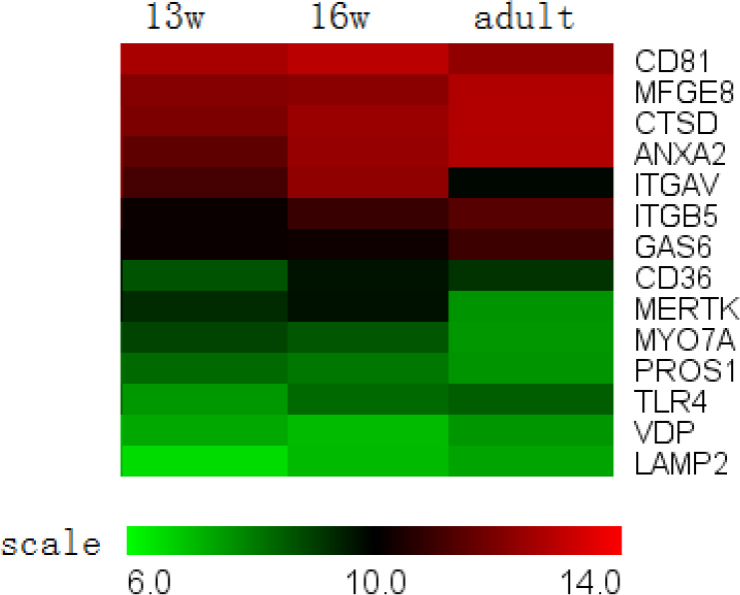
Phagocytic pathway genes expressed in the retinal pigment epithelium. The majority of genes showed constant expression levels throughout development. The expression of *MERTK* and *ITGAV* (integrin αν) decreased during development, but the magnitude of the changes was not very large; therefore, these genes did not appear in our differentially expressed gene list. The only differentially expressed gene was *MYO7A*, the expression of which decreased during development.

**Figure 5:**
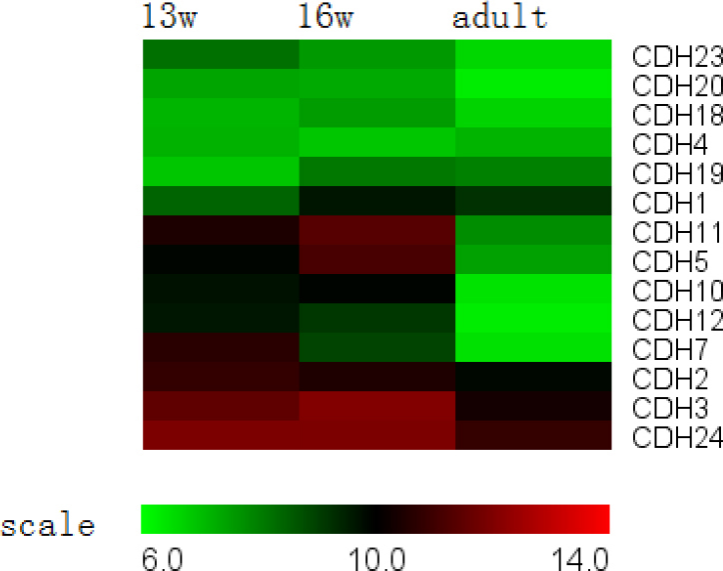
Adherens junction genes expressed in the retinal pigment epithelium. The expression of *Cadherin-7*, *10*, *12*, and *24* was significantly downregulated throughout development, while other cadherin family members exhibitedstable expression.

**Figure 6:**
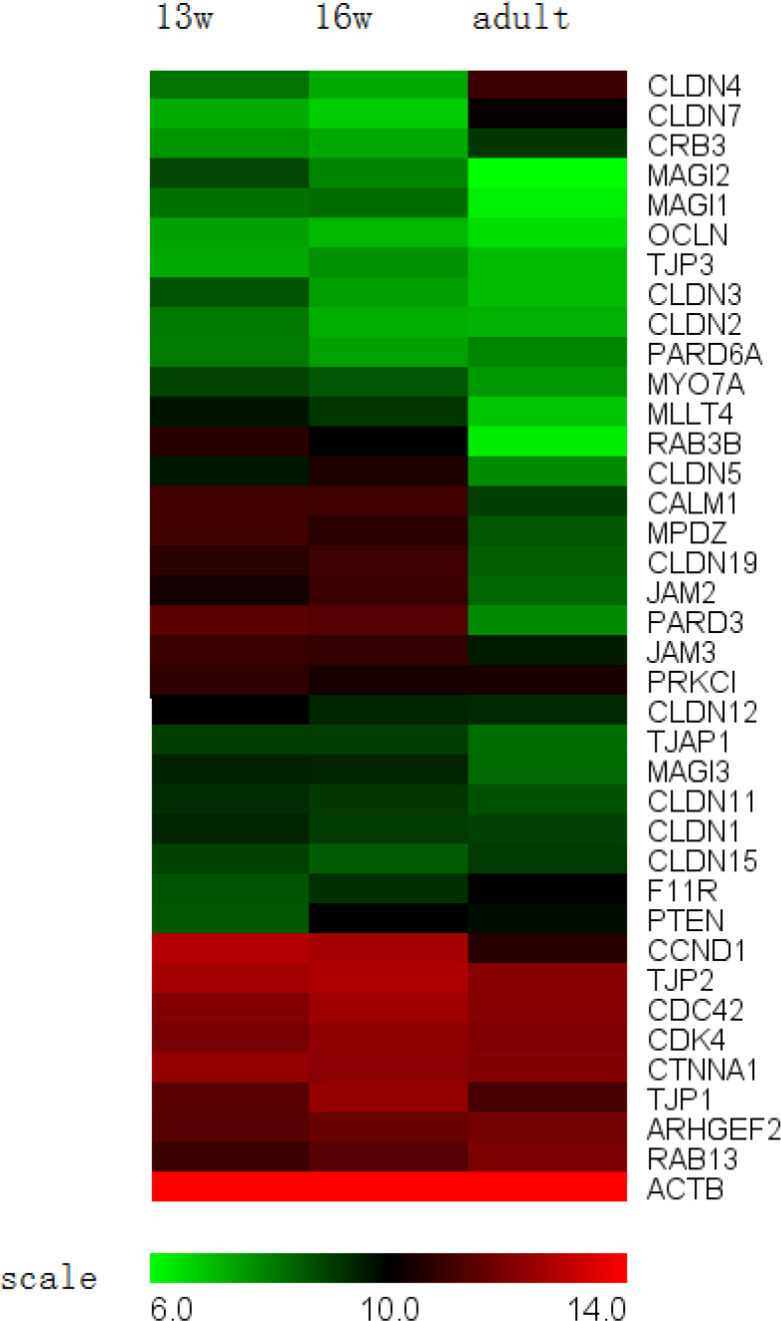
Tight junction genes and known regulators expressed in the retinal pigment epithelium. Some mRNAs (mRNAs) for transmember proteins were stably expressed during development, such as *F11R*, *JAM2*, *CRB3*, and *occludin*. *Claudin-1, 2*, *3*, *5*, and *19* presented a trend of downregulation during development, and *claudin-4* and *7* presented an upregulation trend during development. *JAM-C* expression decreased during development. Genes coding for adaptor proteins, such as *ZO-1*, *ZO-2*, *ZO-3*, *TJAP1*, *MPDZ*, *MAGI1*, *MAGI3*, *ACTB*, and *CTNNA1* (α-catenin), were stably expressed during development. The expression of *MAGI2*, *PARD3*, and *MLLT4* was significantly downregulated between 13-week-old fetuses and adults. Among the effector proteins analyzed, the expression of *CDK4*, *cyclin D1*, and *RAB3B* decreased during development.

**Figure 7:**
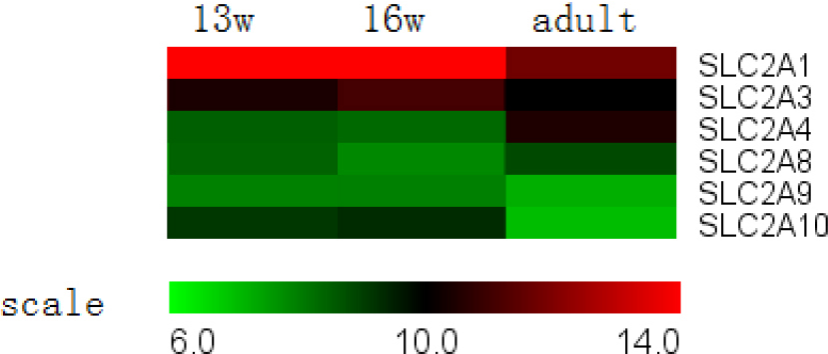
Glucose transporter genes expressed in the retinal pigment epithelium. Some mRNAs (mRNAs) for glucose transporters were stably expressed during development, such as *GLUT3 (SLC2A3)*, *GLUT 4 (SLC2A4)*, *GLUT 8 (SLC2A8)*, and *GLUT 9 (SLC2A9)*, while the expression of *GLUT1 (SLC2A1)* and *GLUT 10 (SLC2A10)* decreased during development.

**Figure 8:**
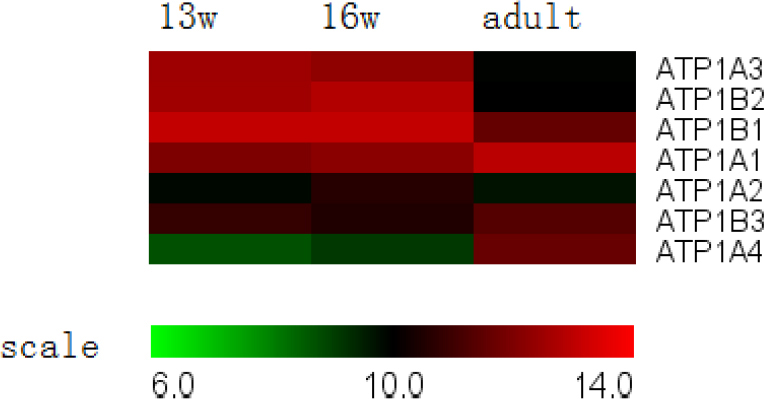
Expression of Na-K-ATPase genes in the retinal pigment epithelium. The majority of genes exhibited constant expression levels throughout development, although the expression of*ATP1B1* decreased during development.

**Figure 9:**
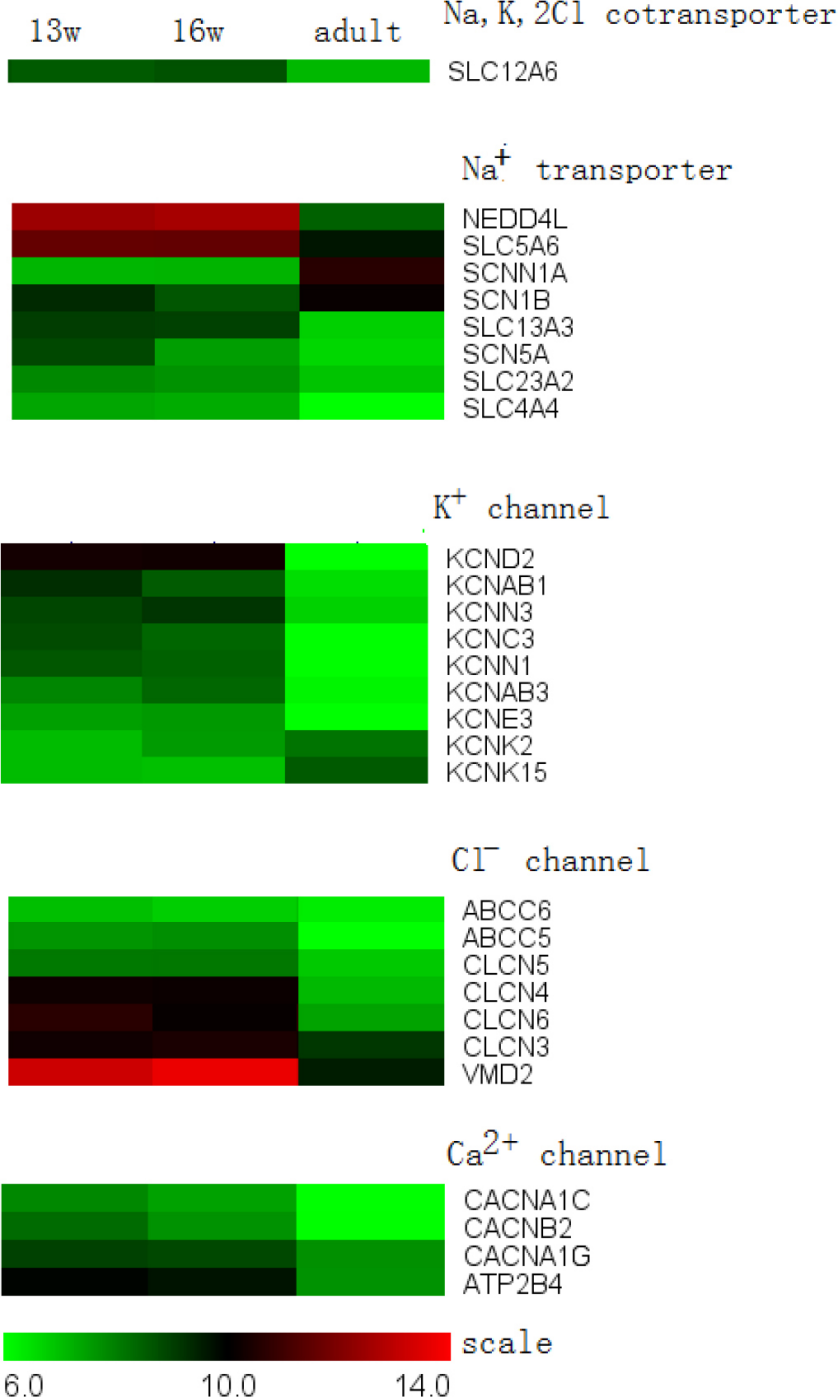
Ion transport protein genes expressed in the retinal pigment epithelium. Several candidate transporters predicted by physiologists were identified. We only listed some of the genes that are differentially expressed during development. None of groups of genes (Na^+^, K^+^, Cl^-^, or Ca^2+^) showed a consistent expression pattern.

With the aim of better characterizing the expression patterns of genes with critical functions within the retinal pigment epithelium, we gathered information on genes grouped by function, namely pigment synthesis, visual cycle, phagocytosis, adherens and tight junctions, and transcellular transport. Genes in these five groups were analyzed, and the results are shown in Figure 2, Figure 3, Figure 4, Figure 5, Figure 6, Figure 7, Figure 8, and Figure 9.

#### I. Pigment synthesis

The pigment synthesis pathway is involved in eumelanin synthesis within melanosomes and melanosomal transporters. Ten genes were identified as participating in RPE pigment synthesis (Figure 2). All of the genes in this group exhibited significant downregulation during development. The gene with the largest decrease was *TYR* (140X), which promotes the first two rate-limiting steps of melanin synthesis.

#### II. Visual cycle

This pathway serves to regenerate or better reisomerize the all-*trans*-retinal complex into the 11-*cis*-retinal complex. The reisomerization occurs in the RPE. Several reviews have described the chemistry and enzymology of the visual cycle in detail [23,24]. As shown in Figure 3, several genes in the visual cycle were stably expressed during development, including *ABCA4*, *RBP1*, *RBP2*, *RBP5*, *RLBP1* (CRALBP*)*, *CRABP1*, and *RDH11*. While other genes in this group showed minimal changes between 13- and 16-week fetuses, a significant decrease in expression was observed between the 16-week-old fetuses and adults, such as *RPE65*, *LRAT*, *ALDH1A2*, *RDH5*, and *RDH14*. The gene with the largest decrease in expression was RPE65 (60X), which participates in a rate-limiting step of the visual cycle. *IRBP* could not be detected in our microarray.

#### III. Phagocytosis

Some of the molecular mechanisms responsible for the phagocytosis of photoreceptor outer segments by the RPE have been examined recently, and several genes implicated in the three stages of RPE phagocytosis (recognition, ingestion, and digestion) have been identified [25]. All of the genes that have been reported to be associated with phagocytosis were present in 13-week-old fetuses. The majority of genes exhibited constant expression levels throughout development (Figure 4). The expression of *MERTK* and *ITGAV* (integrin αν) decreased during development; however, because the magnitude of the changes was not very great, these genes did not appear in our differentially expressed gene list. The only differentially expressed gene was *MYO7A*, which is responsible for the rapid relocation of phagosomes from the apical processes back to the base of the RPE cells [26], and its expression decreased during development.

#### IV. Adherens and tight junctions

Adherens junctions were present before tight junctions and initial cell-to-cell contacts began to form. The cadherin and catenin families control the formation, maintenance, and function of adherens junctions [27]. The expression of *Cadherin-7*, *10*, *12*, and *24* was significantly downregulated throughout development, while other cadherin family members exhibited stable expression (Figure 5). None of the catenin family members exhibited differential expression during development.

Tight junctions are composed of a growing list of proteins that can be grouped into transmembrane, adaptor, and effector proteins [28] (Figure 6). Many of the typical transmembrane proteins, such as F11R, JAM2, JAM3, CRB3, occludin, and claudins 1–5, 7, 11, 12, 15, and 19, were present in our microarray. Claudins play an important role in determining tight junction selectivity and permeability [29], and they undergo constant remodeling during development [30]. In our microarray, *claudin-1, 2*, *3*, *5*, and *19* exhibiteda trend of downregulation, and *claudin-4* and *7* presented an upregulation trend during development. We also found that the expression level of *claudin-19* was the most prominent throughout development compared with the expression levels of the other proteins evaluated, which was in accordance with previous reports [22,31]. As described in the literature, *claudin-19* is an essential component of human RPE tight junctions, and the absence of functional *claudin-19* results in a loss of transepithelial electrical resistance (TER) in culture and severe visual defects in patients [32,33]; therefore, we performed an additional real-time RT–PCR to validate the expression pattern of *claudin-19*. The results of the RT–PCR were in agreement with those of the microarray in that the expression levels of *claudin-19* continuously decreased between 13 weeks of gestation to adulthood. Because the human fetal RPE is a *claudin-19*-dominant epithelium [32], it is reasonable to believe that the remodeling of tight junctions occurred in conjunction with the change in *claudin-19* expression. *JAM-C* exhibited a threefold higher expression intensity in the 13-week-old fetus compared with the 16-week-old fetus. The higher expression of *JAM-C* in the developing RPE is important, as this protein has been implicated in the establishment of the earliest cell-to-cell contacts and in cell polarization [34].

Genes coding for adaptor proteins such as *ZO-1*, *ZO-2*, *ZO-3*, *TJAP1*, *MPDZ*, *MAGI1*, *MAGI3*, *ACTB*, and *CTNNA1* (α-catenin) were present in the fetuses and showed no changes in expression levels compared with those present in the adults. These adaptor proteins are important components of the tight junction complex structure for two reasons: first, they anchor the junction to the cytoskeleton, and second, they may help to regulate the overall function of the structure [35]. The expression of *MAGI2*, *PAR-3 (PARD3)*, and *AF-6 (MLLT4)* was significantly downregulated from 13 weeks of gestation to adulthood. Effector proteins include different types of signaling components, such as GTP-binding proteins, protein kinases, and phosphatases [28]. Among these effector proteins, cell cycle regulators such as *CDK4* and *cyclin D1* showed significant downregulation during development, suggesting that the cell cycle rate was slowed. Another gene exhibiting higher expression in 13-week-old fetuses compared with adults was RAB3B, which is responsible for polarized vesicle transport.

#### V. Transcellular transport

To satisfy the retina’s large requirement for glucose, the RPE expresses high levels of glucose transporters (Figure 7). The current study demonstrated that the housekeeping transporter *GLUT3 (SLC2A3)* was stably expressed, but that *GLUT1 (SLC2A1)* was downregulated during development. There was also stable expression of the mRNA of *GLUT 4 (SLC2A4)*, *GLUT 8 (SLC2A8)*, and *GLUT 9 (SLC2A9)*, whereas the expression of *GLUT 10 (SLC2A10)* continuously decreased from the 13-week-old fetus to adulthood.

Na-K-ATPase, which is located in the apical membrane, provides the energy for transepithelial transport. Functional Na-K-ATPase is a heterodimeric protein consisting of an α-subunit and a β-subunit [36]. Four isoforms of the α-subunit (*α1*, *α2*, *α3*, and *α4*) and three isoforms of the β-subunit (*β1*, *β2*, and *β3*) have been identified and were examined in this study, but only the mRNA expression of the *β1*-subunit decreased during development (Figure 8). The precise role of the β-subunit is still not known, but there is some evidence that the β-subunit may be important for cell-to-cell adhesion [37].

The RPE establishes a polarized distribution of ion pumps, channels, and transporters to mediate the active transcellular transport of ions and organic solutes. Although many transporters have already been identified as highly enriched in the human RPE [38], a genome-wide analysis has not yet been performed. Figure 9 shows some of the genes that are differentially expressed during development. The microarray data revealed thatnone of these groups (Na^+^, K^+^, Cl^-^, and Ca^2+^) exhibiteda consistent expression pattern, and there were often multiple candidates for each given transporter. Furthermore, for a given transporter, the expression of one candidate may have been upregulated when that of another candidate may have been downregulated or stable. The data also suggested the existence of more transporters than have been characterized before. There are two genes of interest: *Bestrophin (VMD2)* and *CFTR (ABCC5*, *ABCC6)*. These two genes both encode chloride channels, and they are found in the basolateral membrane of the RPE. Genetic defects in *bestrophin* or *CFTR* result in Best vitelliform macular dystrophy [39] or reducedamplitudes of the fast oscillation in the EOG [40]. *Bestrophin* and *CFTR* were highly expressed in fetuses compared with adults, which could point to their greater importance in fetuses.

### The expression patterns of retinal pigment epithelium signature genes

A recent comprehensive study comparing the gene expression profiles of the fetal and adult RPE with somatic tissues identified 154 signature genes that are unique to the RPE [22]. Our cross-reference analysis showed that 148 of the candidate signature genes found through their Affymetrix platform could be identified in our NimbleGen microarray. Among the 148 genes, 53 were downregulated, while only 5 were upregulated from the 13-week-old fetus to adulthood. This finding is consistent with the general gene expression pattern that the majority of expressed genes in RPE exhibited, i.e., a trend of downregulation during development. (Fifty-eight differentially expressed genes are listed in Table 2 and Table 3.) There were no differentially expressed genes present between 13 and 16 weeks of gestation. Gene ontology analysis indicated that 53 downregulated genes were involved in transport, eye development, cell cycle, metabolism, protein translation, signal transduction, visual perception, stress responses, inflammation, transcription regulation, pigment synthesis, cell adhesion and matrix biogenesis. The five upregulated genes were *AHR*, growth arrest specific 1 (*GAS1*), *GRAMD3*, *LGALS8*, and *LSR*. Gene ontology analysis revealed that these genes were associated with the cell cycle, signal transduction, embryonic development, and stress responses. It is worth mentioning that *GAS1* is an important transcriptome for the negative regulation of the S phase mitotic cell cycle, and the disruption of *GAS1* results in ectopic RPE proliferation and transdifferentiation in the retina [41]. The expression of *GAS1* increased in the RPE during development, and it was expected that, subsequent to the initial establishment of the RPE in the optic vesicles, proliferation in the presumptive RPE would cease, leading to the formation of a single layer of cuboidal cells. Our results further highlight the important role of upregulated *GAS1* in normal RPE development. Among these 148 signature genes, we found that 90 exhibited minimal changes in expression that varied less than twofold. These genes included many common housekeeping genes, and they were not clustered into any of the expression patterns. Although these genes were less interesting from the standpoint of differential expression analysis, they are a key validation component of the data.

**Table 2:** 58 differentially expressed signature genes during development.

RefSeq ID	Gene symbol	gene name	cluster
NM_001621	*AHR*	aryl hydrocarbon receptor	12
NM_005166	*APLP1*	amyloid beta (A4) precursor-like protein 1	7
NM_015161	*ARL6IP*	ADP-ribosylation factor-like 6 interacting protein	7
NM_004315	*ASAH1*	N-acylsphingosine amidohydrolase (acid ceramidase) 1	7
NM_020139	*BDH2*	3-hydroxybutyrate dehydrogenase, type 2	7
NM_001202	*BMP4*	bone morphogenetic protein 4	7
NM_001801	*CDO1*	cysteine dioxygenase, type I	7
NM_001830	*CLCN4*	chloride channel 4	7
NM_000743	*CHRNA3*	cholinergic receptor, nicotinic, alpha 3	7
NM_005202	*COL8A2*	collagen, type VIII, alpha 2	9
NM_006574	*CSPG5*	chondroitin sulfate proteoglycan 5 (neuroglycan C)	7
NM_001329	*CTBP2*	C-terminal binding protein 2	7
NM_177538	*CYP20A1*	cytochrome P450, family 20, subfamily A, polypeptide 1	7
NM_015115	*DCUN1D4*	DCN1, defective in cullin neddylation 1	9
NM_001930	*DHPS*	deoxyhypusine synthase	2
NM_001037954	*DIXDC1*	DIX domain containing 1	2
NM_018100	*EFHC1*	EF-hand domain (C-terminal) containing 1	
NM_001040092	*ENPP2*	ectonucleotide pyrophosphatase/phosphodiesterase 2	7
NM_022970	*FGFR2*	fibroblast growth factor receptor 2	9
NM_002048	*GAS1*	growth arrest-specific 1	
NM_000165	*GJA1*	gap junction protein, alpha 1, 43 kDa	7
NM_018178	*GOLPH3L*	golgi phosphoprotein 3-like	
NM_001001996	*GPM6B*	glycoprotein M6B	2
NM_000273	*GPR143*	G protein-coupled receptor 143	7
NM_023927	*GRAMD3*	GRAM domain containing 3	
NM_025103	*IFT74*	intraflagellar transport 74 homolog (Chlamydomonas)	2
NM_001007225	*IGF2BP2*	insulin-like growth factor 2 mRNA binding protein 2	7
NM_017644	*KLHL24*	kelch-like 24 (Drosophila)	7
NM_006499	*LGALS8*	lectin, galactoside-binding, soluble, 8	

**Table 3:** 58 differentially expressed signature genes during development.

RefSeq ID	Gene symbol	Gene name	cluster
NM_004789	*LHX2*	LIM homeobox 2	7
NM_005576	*LOXL1*	lysyl oxidase-like 1	7
NM_205834	*LSR*	lipolysis stimulated lipoprotein receptor	8
NM_005584	*MAB21L1*	mab-21-like 1 (C. elegans)	7
NM_024641	*MANEA*	mannosidase, endo-alpha	7
NM_144778	*MBNL2*	muscleblind-like 2 (Drosophila)	
NM_021647	*MFAP3L*	microfibrillar-associated protein 3-like	9
NM_015460	*MYRIP*	myosin VIIA and Rab interacting protein	9
NM_014903	*NAV3*	neuron navigator 3	7
NM_015277	*NEDD4L*	neural precursor cell expressed	7
NM_003489	*NRIP1*	nuclear receptor interacting protein 1	7
NM_022062	*PKNOX2*	PBX/knotted 1 homeobox 2	7
NM_002841	*PTPRG*	protein tyrosine phosphatase, receptor type, G	
NM_022337	*RAB38*	RAB38, member RAS oncogene family	7
NM_016026	*RDH11*	retinol dehydrogenase 11 (all-trans and 9-cis)	
NM_000329	*RPE65*	retinal pigment epithelium-specific protein 65 kDa	9
NM_004866	*SCAMP1*	secretory carrier membrane protein 1	7
NM_002998	*SDC2*	syndecan 2	9
NM_003015	*SFRP5*	secreted frizzled-related protein 5	7
NM_013257	*SGK3*	serum/glucocorticoid regulated kinase family, member 3	
NM_005413	*SIX3*	sine oculis homeobox homolog 3	2
NM_182767	*SLC6A15*	solute carrier family 6, member 15	7
NM_003051	*SLC16A1*	solute carrier family 16, member 1	7
NM_012319	*SLC39A6*	solute carrier family 39 (zinc transporter), member 6	7
NM_015464	*SOSTDC1*	sclerostin domain containing 1	7
NM_005843	*STAM2*	signal transducing adaptor molecule	
NM_014640	*TTLL4*	tubulin tyrosine ligase-like family, member 4	7
NM_000550	*TYRP1*	tyrosinase-related protein 1	7
NM_004183	*VMD2*	Best 1, bestrophin 1	7

### Validation of gene expression with qualitative real-time reverse transcriptase–PCR

**Figure 10:**
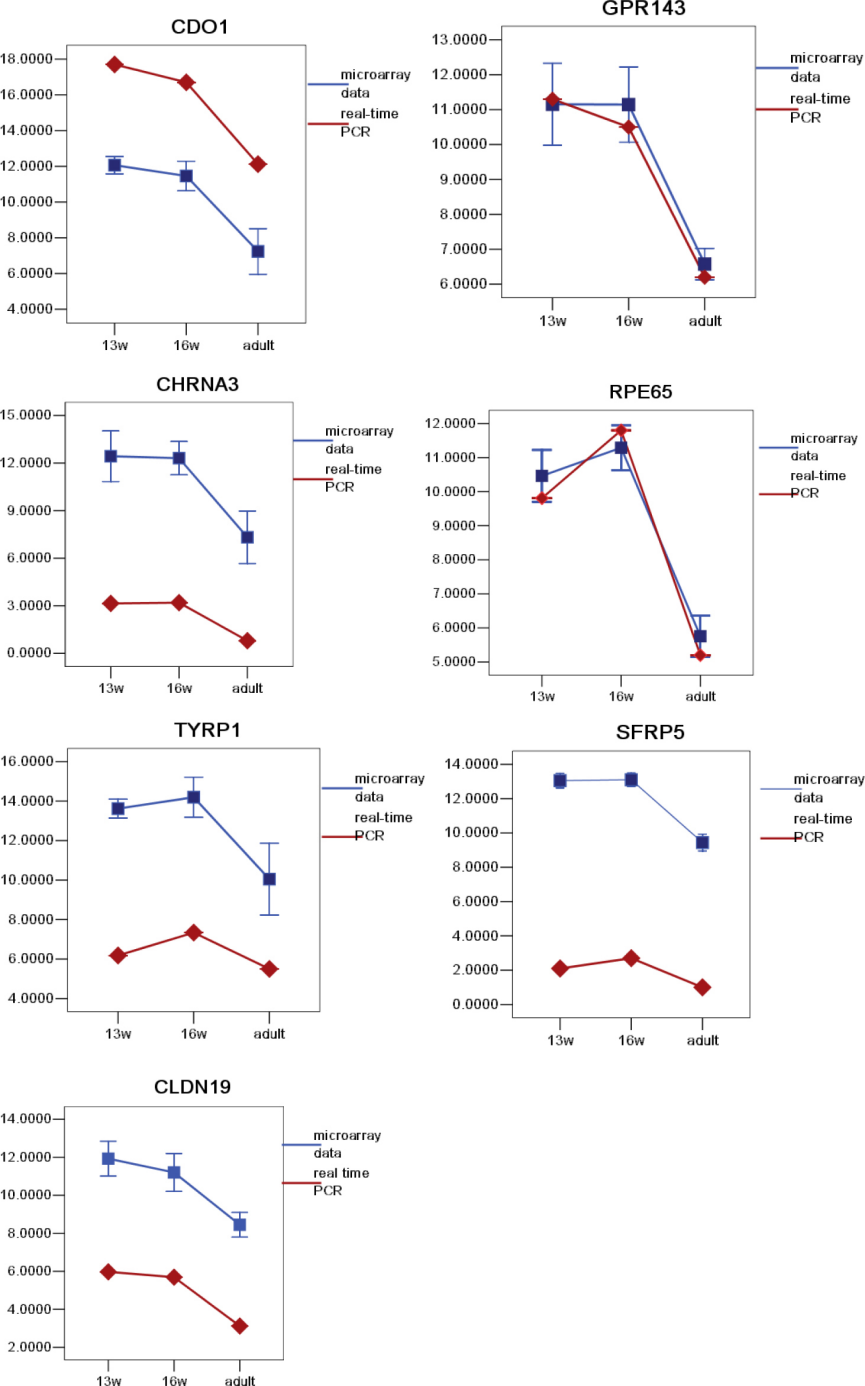
Validation of the microarray gene expression profiles. The expression profiles of *CDO1*, *GPR143*, *CHRNA3*, *RPE65*, *TYRP1*, *SFRP5*, and *CLDN19* were validated by comparing the microarray data with the gene expression data from real-time RT–PCR. Various profiles were represented, and in each case, the data from the microarray analysis closely resembled the data generated by real-time RT–PCR.

Seven genes that exhibited high degreesof differential expression in the microarray analysis or that are functionally important to the RPE (*CDO1*, *GPR143*, *CHRNA3*, *RPE65*, *TYRP1*, *SFRP5*, and *CLDN19*) were chosen for further validation by qRT^2^-PCR analysis. As shown in Figure 10, the real-time RT–PCR results conﬁrmed the microarray data and showed similar expression trends to those observed from the microarray analysis at the three time points. However, the fold changes determined by the two techniques varied. For example, for *CHRNA3* and *SFRP5*, the microarray analysis showed 35- and 16 fold downregulations after normalization by the glyceraldehyde-3-phosphate dehydrogenase housekeeping gene, respectively; the real-time PCR analysis showed only 15- and eightfold changes, respectively.

## Discussion

Although the RPE is fundamentally important to retinal development and function and it is a critical focus of retinal degenerative diseases and therapeutic interventions, relatively little is known about the molecular mechanisms of human RPE development. This is the first study to demonstrate gene expression profiles in the human RPE during normal development. Approximately 4 weeks into human embryogenesis, the neural ectoderm grows out from the diencephalons to form the optic vesicle; it theninvaginates to form the optic cup. The outer layer of the optic cup becomes the RPE, and the inner layer becomes the neural retina. Melanin synthesis starts as early as 35 days of gestation, and RPE cells become full of pigment in the following weeks [42]. Consistent with minor changes in morphology, only 10% of the RPE genes showed differential expression between 13 weeks of gestation and adulthood. It appears that genes with consistently decreasing expression during RPE development are more common than genes with consistently increasing expression.

The RPE is an unusual epithelium, and it performs numerous functions that are essential for visual function. To better characterize the expression patterns of genes with critical functions within the RPE, we also performed a more focused analysis of the expressed genes grouped by function. All of the genes associated with pigment synthesis exhibited consistently decreasing expression between the fetuses and adults. This finding is in line with previous studies [43], which found that the majority of the melanin pigment in the RPE is synthesized prenatally within a few weeks of embryonic development, and it is stored in the melanosomes for the entire lifespan of the individual. Age, various types of light damage, and oxygen free radicals can contribute to the reduction of melanosomes in adults. It is known that melanin can exert a protective function in RPE cells in several ways. First, melanin shields these cells from the damage induced by sunlight and ultraviolet radiation [44]. Second, melanin can counteract the oxidative stress caused in the RPE by free radicals derived from lipid peroxidation products [45] and by accumulated iron [46]. Third, the high binding capacity of melanin to metal ions [47] and exogenous chemicals [48] also lends support to the protective role of melanin in the eye. A reduced capability to absorb light energy and increases in oxidative stress with aging are also important factors in the cascade of events leading to AMD, which may be the reason that AMD is more common in elderly people. The expression levels of genes involved in the visual cycle exhibited a downregulation trend. This finding is somewhat surprising because, with the formation of the photoreceptor outer segments, one would expect upregulation of visual cycle protein expression, as has been found in chickens and rodents [20,49]. By analyzing the expression levels of genes associated with phagocytosis, we found that there was also no expected upregulation trend with the formation of the outer segments and the lengthening of the RPE microvilli, but there was instead a slight decrease with development. To further validate the expression patterns of genes involved in the visual cycle and in phagocytosis in the human RPE, we compared our data with previous experimental results. Miller’s laboratory identified a set of genes that are unique to the RPE using a microarray technique [22]. Among these genes, three (*RPE65*, *RBP1*, and *RDH11*) are involved in the visual cycle and four (*ITGAV*, *VDP*, *LAMP_2_*, and *GULP1*) are involved in phagocytosis. *RPE65*, *RBP1*, *ITGAV*, and *GULP1* exhibited higher expression levels in the native fetal RPE compared with the native adult RPE. The expression levels of *RDH11*, *VDP*, and *LAMP_2_* remained relatively unchanged between native fetuses and native adults. It was reported that the outer segments of the cones and rods are not fully developed until the 30^th^ week of gestation [50]. All of these results support the hypothesis that the expression of genes associated with the visual cycle and phagocytosis is not coordinated with the morphological maturation of the outer photoreceptor segments.

The apical junctional complex, composed of adherens and tight junctions, is an integrated signaling complex that helps to regulate cell shape, polarity, and proliferation [27]. Most of the proteins found in all epithelial cells were presented in our microarray. These proteins form a complicated interacting network and are often regulated by posttranslational modifications. Therefore, it is difficult to correlate the expression levels of a handful of mRNAs with biologic processes. However, there were several genes of interest. *Claudin-19* showed minimal changes in expression between 13 and 16 weeks of gestation but significantly decreased expression between 16-week-old fetuses and adults. As *claudin-19* is the predominant claudin in the human RPE [32], changes in the expression level of claudin-19 may indicate changes in the selectivity and permeability of tight junctions. It is speculated that a relatively static extracellular environment is needed for embryogenesis because the development of the neural retina occurs slowly and the visual neurophysiology is inactive during embryogenesis. The higher expression of *claudin-19* in the fetal RPE is thought to result in the formation of more “tight” junctions and a more powerful outer blood-retinal barrier, with greater selectivity and lower permeability. This hypothesis was supported by our previous study, in which we found that the mRNA of *claudin-19* was more prominent than other claudins in cultured human RPE cells isolated from fetal eyes.

The TERs of hfRPE were approximately 1,000 and 400 Ω×cm^2^ in serum medium and serum-free medium, respectively [32,51], which is much higher than the value of 206 Ω×cm^2^ that was estimated for the adult human RPE in vivo [52]. The permeability of the barrier increased 10 fold with the knockdown of *claudin-19* by small interfering RNA [32]. There are two reasons for the decreasing expression of *claudin-19* in adults: on one hand, increased barrier permeability is needed with increasing activity of the visual neurophysiology and the increased requirements of nutrients and ions; on the other hand, proinflammatory cytokines increase junction permeability via decreases in the expression of *claudin-19* [51]. As another example, the TER in the ARPE-19 cell line, which was transformed from adult cells without the expression of *claudin-19*, was found to be approximately 50 Ω×cm^2^ [52]. The expression of *JAM-C*, *AF-6*, and *PAR-3* was consistently downregulated in the 13-week-old fetus. As described in the literature, *JAM*, *AF-6*, and the *PAR-3/PAR-6/aPKC* complex play major roles in the initial formation of adherens junctions and contribute to cell polarity [53], and the expression of *JAM* and *AF-6* decreased substantially after the formation of adherens junctions. Thus, it is reasonable to believe that adherens junctions and cell polarity are established before 13 weeks of gestation in the human RPE.

The expression of many membrane pumps, channels, and transporters is up- or downregulated during human RPE development. A few observations are notableupon comparison of our results with those of earlier studies on the developing chicken RPE. As tight junctions become “tighter” and compensate for the decrease in the paracellular pathway, the expression of glucose transporters is upregulated during chicken RPE development [54]. However, glucose transporters in the human RPE follow the opposite developmental pattern; that is, the expression is downregulated during development. It is unclear whether this downregulation is coordinated with tight junction permeability, but there must be a different regulation mechanism between chickens and humans. Another remarkable gene was *Bestrophin*, which was found to be upregulated during chicken RPE development but downregulated during human RPE development.

The results presented here demonstrate that, although the human RPE follows the same pattern of differentiation as that in other vertebrates at the macroscopic level, there are notable differences at the molecular level. In chicks and rodents, the expression of the visual cycle proteins is coordinated with the formation of the outer photoreceptor segments and displays a gradual polarization of the RPE, but the human RPE appears to achieve a high degree of maturity as early as 13 weeks of gestation (corresponding to the early phase of human retinal development). These results are supported by the observation that human RPE cells isolated from 16-week-old fetuses are already highly polarized, with a high TER, and the visual cycle is already well established [55]. The different developmental pattern of the human RPE may be a mechanism to protect the photoreceptors from toxins during the long period of gestation.

Recently, the molecular signature of the human RPE was identified, and 154 RPE signature genes were validated by microarray and qRT^2^-PCR analysis, both in the RPE and in an independent set of 78 tissues [22]. We cross-referenced our data with this reported data set for two reasons: first, we wanted to validate the accuracy of our microarray data; and second, this well defined data set enabled easier identification of important regulators during RPE development. Using their identified signature gene list, we found 58 genes showing twofold or more differences in expression at the three examined time points. These genes participate in a wide spectrum of activities, such as transport, eye development, the cell cycle, metabolism, signal transduction, visual perception, and pigment synthesis, suggesting that these 58 genes are the core developmental genes in the human RPE.

The present study not only offers information on the trends of changes in gene expression levels during human RPE development, but has also identified a set of genes that are crucial for developmental regulation. These results may be helpful in furthering our understanding of the developmental processes of the human RPE.
